# Acute influenza virus-associated encephalitis and encephalopathy in adults: a challenging diagnosis

**DOI:** 10.1099/jmmcr.0.005076

**Published:** 2016-12-19

**Authors:** Wouter J. Meijer, Francisca H. H. Linn, Anne M. J. Wensing, Helen L. Leavis, Debby van Riel, Corine H. GeurtsvanKessel, Mike P. Wattjes, Jean-Luc Murk

**Affiliations:** ^1^​ Perinatal Center, Wilhelmina Childs Hospital, University Medical Center Utrecht, KE 04.123.1, Lundlaan 6, PO Box 85090, Utrecht, The Netherlands; ^2^​ Department of Neurology and Neurosurgery, Brain Center Rudolf Magnus, University Medical Center Utrecht, Room G03-228, Heidelberglaan 100, 3584 CX Utrecht, The Netherlands; ^3^​ Department of Medical Microbiology, University Medical Center Utrecht, Heidelberglaan 100, 3584 CX Utrecht, The Netherlands; ^4^​ Department of Rheumatology and Clinical Immunology, University Medical Center Utrecht, Heidelberglaan 100, 3584 CX Utrecht, The Netherlands; ^5^​ Department of Viroscience, Erasmus Medical Center, Wytemaweg 80, 3015 CN Rotterdam, The Netherlands; ^6^​ Department of Radiology and Nuclear Medicine, VU University Medical Center, De Boelelaan 1117, 1081 HV Amsterdam, The Netherlands

**Keywords:** MRI, oseltamivir, corticosteroids., cerebrospinal fluid

## Abstract

**Background**: Acute influenza-associated encephalopathy/encephalitis (IAE) in adults is a rare but well-known complication of influenza virus infection. The diagnosis is difficult to make due to the absence of distinctive clinical symptoms and validated diagnostic criteria. We present an illustrative case and a case review on acute IAE in adults.

**Methods**: We performed a Medline search of the English literature using the terms influenz*, encephal* and adult, and constructed a database of detailed descriptions of patients with influenza virus infection with influenza-like symptoms at the onset of neurological symptoms.

**Results**: A total of 44 patients were included. Confusion and seizures were the most prevalent neurological symptoms, present in 12 (27 %) and 10 (23 %) patients, respectively. Magnetic resonance imaging (MRI) was performed in 21 patients and anomalies were found in 13 (62 %), with lesions located throughout the brain. Influenza virus RNA was detected in cerebrospinal fluid (CSF) in 5 (16 %) of 32 patients. Eight (18 %) of the forty-four patients died. The benefits of antiviral and immunomodulatory therapy have not been well studied.

**Discussion**: Our results show that many different neurological symptoms can be present in patients with acute onset IAE. Therefore, the diagnosis should be considered in patients with fever and neurological symptoms, especially during the influenza season. Laboratory diagnosis consists of demonstration of influenza virus RNA in brain tissue, CSF or respiratory samples, and demonstration of intrathecal antibody production against influenza virus. The presence of brain lesions in MRI and influenza virus in CSF appear to be of prognostic value.

## Introduction

Neurological complications of influenza virus infection are a rare but well-known cause of morbidity and mortality in children and adults worldwide, and their incidence appears to have increased after the 2009 H1N1 influenza A virus pandemic (Glaser *et al.*, 2012; Gu *et al.*, 2013; Hjalmarsson *et al.*, 2009). Influenza-associated encephalopathy/encephalitis (IAE) is a neurological condition associated with influenza virus infection. The diagnosis is difficult because there is no uniform clinical presentation, influenza virus is rarely detected in cerebrospinal fluid (CSF) and may no longer be detectable in respiratory samples when the patient presents with neurological symptoms. Furthermore, validated diagnostic criteria for this condition in adults are lacking. Thus, extensive clinical tests are needed to exclude other causes of encephalopathy, before the presumptive diagnosis of IAE is made. To highlight the complicated and challenging diagnostic process, we present the case of a patient with acute onset IAE, who had a second episode of acute onset IAE 22 months later. In addition, after a comprehensive review of the literature of IAE in adults, we propose a diagnostic algorithm to aid clinicians in early and correct diagnosis.

## Case report

In February 2013, a 58-year-old man was admitted to the University Medical Center Utrecht, The Netherlands, due to acute onset confusion. He had a cough and had felt ill and been febrile for the past 3 days. He also had a headache, a decreased appetite and was restless and agitated. His past medical history did not reveal any remarkable features. He had not received an influenza vaccine in the previous 6 months. On the day of admission he was confused, and used incorrect words and sentences. On physical examination no abnormalities were noted, apart from a temperature of 38.1 °C (measured in the ear). Neurological examination showed an alert patient with apraxia and global dysphasia with paraphasia, but in the hours after admission he gradually developed mutism. Neither meningism nor cranial nerve dysfunction were present. The patient was admitted to the neurology ward with a provisional diagnosis of infectious encephalitis. Extensive investigations were performed (Table 1), and empiric treatment with acyclovir 10 mg kg^−1^ t.i.d., amoxicillin 2 g q.4.h. and ceftriaxone 2 g b.i.d. was initiated. PCR on a nasopharyngeal swab and culture of sputum and urine only revealed a positive PCR for influenza virus on the nasopharyngeal swab. In the CSF, no microorganisms/viruses, autoantibodies or monoclonal B or T cells could be demonstrated. An electroencephalography (EEG) analysis showed diffuse slowing without epileptiform discharge.

**Table 1.** Summary of investigations

Type of investigation	Analysis	First episode (February 2013)	Second episode (December 2014)
Clinical chemistry at admission	Blood	Leukocytes: 3.8×10^9^ cells l^−1^ (normal range: 4–10×10^9^ cells l^−1^); thrombocytes: 118×10^9^ cells l^−1^ (normal range: 150–450×10^9^ cells l^−1^); no other abnormalities	Haemoglobin: 8.2 mmol l^−1^ (=pre-existent value); leukocytes: 4.7×10^9^ cells l^−1^; thrombocytes: 124×10^9^ cells l^−1^; no other abnormalities
	CSF	Leukocytes: 2×10^6^ cells l^−1^ (normal); protein: 0.92 g l^−1^ (elevated); IgG spectrum – eight bands, identical to serum	Leukocytes: 1×10^6^ cells l^−1^ (normal); protein: 1.42 gram l^−1^ (elevated)
Microbiology	CSF	No pathogens detected with PCR and culture*	No pathogens detected with PCR and culture†
	CSF/serum antibody index serology	IgG influenza A virus Reiber index: 2.93 (borderline elevated), no elevation of antibodies to other viruses; in consecutive CSF samples rise of IgG antibody titre to influenza A virus H3 antigen (before administration of immunoglobulins)	Inconclusive due to high albumin in CSF
	Blood/serology	HIV negative, no signs of *Treponema pallidum* and *Borrelia burgdorferi* infection	HIV negative, no signs of *Treponema pallidum* and *Borrelia burgdorferi* infection
Immunology		No monoclonal B or T cells in CSF; no auto-antibodies or paraneoplastic markers‡	Six months after disease episode – T cells and subsets, normal absolute numbers; B cells 54 cells mm^−3^ (normal range: 114–436 cells mm ^−3^); IgA-total: 0.59 g l^−1^ (normal range: 0.70–4.0 g l ^−1^); IgG-total 16.3 g l^−1^ (normal range: 7.0–16.0 g l^−1^); no particular immunodeficiency identified, except for a monoclonal gammopathy of unknown significance.
Clinical genetics		No abnormalities in RANBP2 and SCN1a genes	

∗Viruses tested: adenovirus, herpes simplex virus 1 and 2, VZV, Epstein–Barr virus, cytomegalovirus, human herpesvirus type 6, influenza virus, enterovirus, parechovirus, Japanese encephalitis virus. Bacteria tested: general culture, *Mycoplasma pneumoniae, Mycobacterium tuberculosis* complex, listeria. Parasites tested: toxoplasma. Fungi tested: *Cryptococcus*.

†Viruses tested: adenovirus, herpes simplex virus 1 and 2, VZV, Epstein–Barr virus, cytomegalovirus, human herpesvirus type 6, human immunodeficiency virus, influenza virus, enterovirus, parechovirus, JC polyomavirus. Bacteria tested: general culture, listeria.

‡Anti-amphiphysin, anti-Hu, anti-CV2, anti-PNMA2, anti-Ri, anti-YO, anti-VGKC (including LG1 and CASPR2), anti-GAD, anti-GBM, anti-TPO and anti-NMDA-receptor antibodies all negative.

**Fig. 1.**
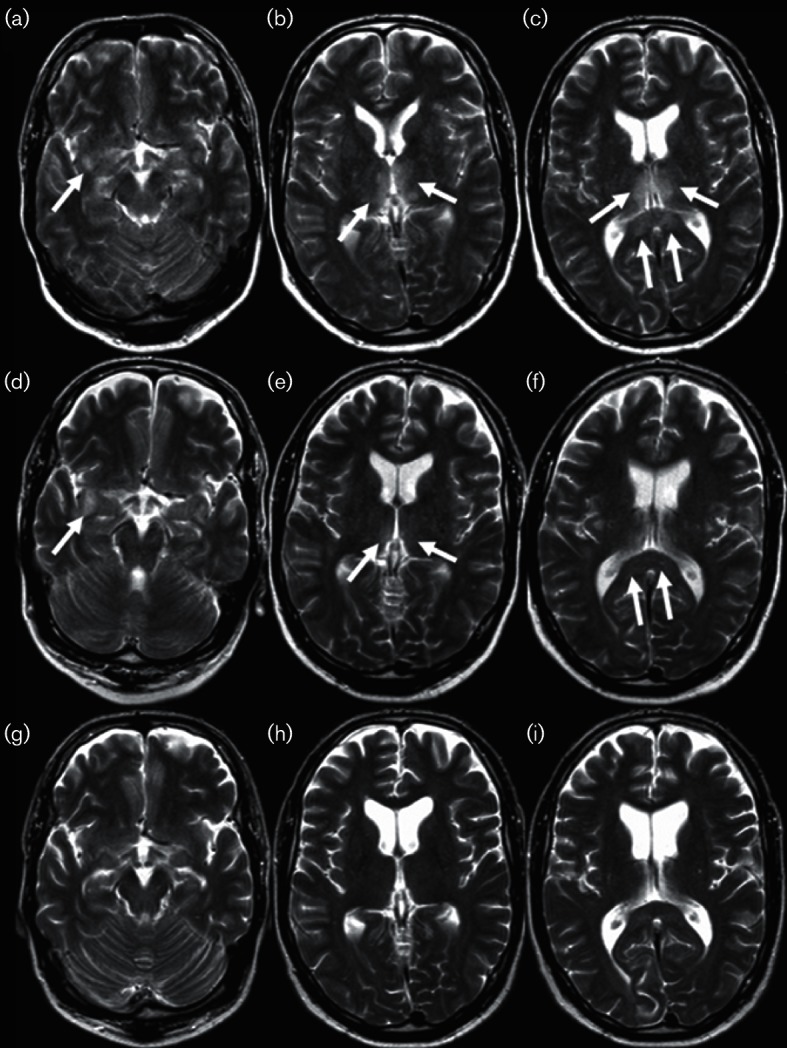
Brain MRI of the patient in our case report. Axial T2-weighted magnetic resonance images at the time of the initial presentation (a–c), 1 week later (d–f) and 2 months later (g–i) demonstrating the distribution and evolution of lesions. On the initial MRI, focal T2-hyperintense lesions were identified in the subcortical white matter of the insula (a, arrow), bilateral in the thalamus (b, arrows; c, top two arrows) and the splenium of the corpus callosum (c, bottom two arrows). One week later the lesions regressed in size. Only the lesion in the insula showed mild progression (d, arrow). On the last follow-up scan 2 months later (g–i), none of the focal lesions were visible anymore. In addition, no neurodegenerative sequelae could be identified.

The patient's level of consciousness gradually deteriorated and he became comatose 2 days after admission. He was transferred to the intensive care unit (ICU) and required mechanical ventilation. A brain magnetic resonance imaging (MRI) scan showed multifocal T2 high signal intensity lesions in grey as well as in white matter, including the ventral corpus callosum without enhancement with gadolinium and without diffusion restriction on diffusion-weighted images (Fig. 1). At that time, the results of the aforementioned microbiological tests became available. Treatment with oseltamivir 150 mg b.i.d. was started and methylprednisolone 1000 mg q.d. was added for 3 days. Thereafter, because the patient was still comatose, treatment with intravenous immunoglobulins 36 g q.d. for 5 days and prednisone 75 mg q.d. then tapered was initiated. A few days later, the condition of the patient gradually improved and 2 weeks after the initial presentation he was discharged from the ICU. The patient’s neurological condition slowly recovered and he was discharged from the hospital 9 weeks after admission. At that time, he suffered from weakness in both hands and lower limbs, and urinary incontinence. On neurological examination, a severe bilateral pyramidal paraplegia, predominantly of the legs, was present with bilateral Babinski signs.

At follow-up 1 year later, the remaining neurological symptoms were weakness of the legs with a pyramidal paraparesis on examination, making the patient wheelchair-bound, and urinary retention, which required self-catheterization. Cognitive functioning was normal.

In December 2014, almost 2 years after the first episode, this patient was readmitted to the hospital with acute onset of aphasia and confusion after several days of influenza-like symptoms. Treatment with oseltamivir 75 mg b.i.d.(initially combined with acyclovir 10 mg kg^−1^ t.i.d.) was initiated. Again, extensive testing was performed (Table 1) and PCR on a nasopharyngeal swab tested positive for influenza virus. Additional analysis revealed a monoclonal gammopathy of unknown significance. In the following days his cognitive functions initially deteriorated until he was mute and apathetic. Fortunately, he gradually recovered 4 days after admission and could be discharged after 12 days without additional sequelae. He now receives yearly influenza vaccination without adverse events.

This case illustrates severe neurological complications during repeated infection with influenza virus. Extensive testing was required to conclude that the patient suffered from IAE. The literature on this topic mainly consists of case reports. We reviewed the literature in order to summarise clinical features, diagnostic tests, treatment options and prognosis in these patients.

## Case review

### Methods

Data for the case review were identified by searching Medline. The search was performed in November 2013 and updated in May 2016. The following search criteria were used: influenz*, encephal* and adult. There were no restrictions on publication dates. References were imported into RefWorks to be further analysed. The search was performed by W. J. M. There were no restrictions on the type of influenza. The search was limited to articles in English. Reports that had not been the subject of peer review, such as abstracts of congress presentations, were excluded. Articles were first screened by title and then by abstract. If they appeared appropriate, a full text evaluation was performed. Articles were added by checking cross-references. We included articles with cases of neurological complications of influenza virus infection. Only cases of patients with influenza-like symptoms present at the onset of neurological symptoms were included. Furthermore, sufficient details of the cases had to be available. Since we intended to study cases of acute onset IAE, we excluded cases with neurological symptoms after prolonged ICU admission for respiratory insufficiency due to influenza virus infection, and cases with influenza virus isolated from the upper respiratory tract but another virus isolated from the CSF. A database of included cases was constructed using Microsoft Excel.

### Results

**Fig. 2.**
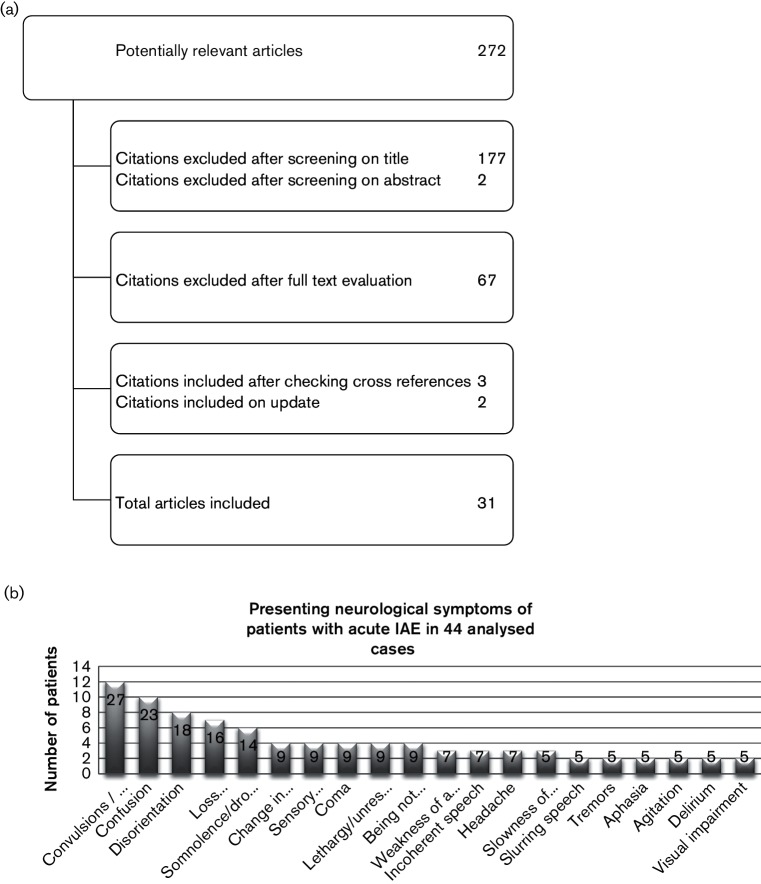
(a) Results of the search. (b) Presenting neurological symptoms of patients with acute IAE in 44 analysed cases. Figures in the bars show the percentage of patients presenting with the symptom. Other symptoms that were reported once were trigeminal neuralgia, restlessness, continuous mumble, memory disturbance, tetraplegia, restlessness, collapse, dysphasia, urinary retention, photophobia, gait disturbance and dizziness. Symptoms are presented as they were described by the authors of the original case reports.

The results of the search are presented in Fig. 2. In total, 44 cases of IAE in adults were identified from 31 reports (Ak *et al.*, 2012; Akins *et al.*, 2010; Alakare *et al.*, 2010; Bartynski *et al.*, 2009; Bulakbasi *et al.*, 2006; Chen *et al.*, 2010; Cheng *et al.*, 2011; Cunha *et al.*, 2012; Fukami *et al.*, 2005; Gadoth *et al.*, 2010; Goenka *et al.*, 2014; Gonzalez & Brust, 2009; Hakoda & Nakatani, 2000; Hawkins *et al.*, 1987; Hjalmarsson *et al.*, 2009; Iijima *et al.*, 2002; Ishii *et al.*, 2015; Ito *et al.*, 2011; Jeganathan *et al.*, 2013; Khosla *et al.*, 2011; Kimura *et al.*, 2008; Lee *et al.*, 2010; Salonen *et al.*, 1997; Santini *et al.*, 2012; Simon *et al.*, 2013; Steinberg *et al.*, 1972; Steininger *et al.*, 2003; Tsai *et al.*, 2011; Wang *et al.*, 2011, 2015; Yamashita *et al.*, 2008). The cases are summarized in the supplementary material. The majority of the patients were males (30/44, 68 %). The median age of the patients was 46 years (range 20–86 years). Except for two patients with a history of a brain infarction, none of the patients had a history of neurological diseases. Five patients had diabetes. There were no patients with an underlying immune disease. One patient used hydrocortisone for panhypopituitarism. One patient was pregnant. Five had alcoholic liver disease or a history of alcohol abuse. Information on influenza vaccination was available for 15 patients of whom 2 (13 %) were vaccinated.

On admission, more than 30 different neurological symptoms were reported (Fig. 2). Convulsions/seizure and confusion were the most prevalent symptoms, present in 27 and 23 % of cases, respectively. All but three patients (93 %) had fever at presentation.

Blood analysis was available for 28 of 44 patients reviewed and was abnormal in 20 (71 %). Thrombocytopenia, an elevated C-reactive protein and leukocytosis were the most common abnormalities, and were present in nine, nine and eight patients, respectively. In these patients, platelet counts ranged from 25 to 39×10^9^ platelets l^−1^ (median 110×10^9^ platelets l^−1^), while the C-reactive protein values ranged from 7 to 343 mg l^−1^ (median 41 mg l^−1^) and leukocyte counts from 11 to 18×10^9^ cells l^−1^. MRI was performed in 21 cases. In 13 it was abnormal, including multiple lesions in 10 cases and a single lesion in 3 cases. Brain oedema was observed in five cases [either by computed tomography (CT) or MRI]. EEG was performed in 25 cases. It was abnormal in 15 cases (60 %), mostly described as generalized or diffuse slowing (9 cases) or consistent with encephalitis (4 cases). CSF analysis was described in 38 cases and was abnormal in 26 (68 %). An elevated white blood cell count and/or an elevated protein concentration were observed most frequently, in 16 and 9 patients, respectively. Influenza virus RNA in CSF was detected in 5/32 cases (16 %). In a single case, influenza virus RNA was detected in a brain biopsy specimen, obtained after the patient died.

Data on treatment was available in 33 cases. Two patients did not receive any medication, while thirty-one were treated with (a combination of) different drugs. The most-prescribed drugs were oseltamivir for 25 patients, acyclovir for 13 patients, broad-spectrum antibiotics for 15 patients and corticosteroids for 9 patients. Gamma-globulins, lanamivir, peramivir and amantadine were also infrequently used (in two, one, one and one patient, respectively). In addition, anti-epileptic drugs (fosphenytoin, levetiracetam), propofol, midazolam, haloperidol and chlorpromazine were administered to treat different neurological/psychiatric symptoms, like epilepsy and delirium. The majority of patients (27/44, 61 %) showed a full recovery, whereas 9 (20 %) had sequelae and 8 (18 %) patients died. Of those who recovered, 21/27 (78 %) recovered within 2 weeks from the onset of symptoms.

## Discussion

Neurological complications of influenza virus infections are relatively rare and mainly affect children (Gu *et al.*, 2013). The incidence is hard to establish since different definitions exist, but has been estimated at 0.21 per million population per year (Hjalmarsson *et al.*, 2009). The incidence of IAE appears to have increased after the 2009 H1N1 influenza A virus pandemic, with an estimated 12 per million of symptomatic 2009 H1N1 cases (Glaser *et al.*, 2012; Gu *et al.*, 2013). In studies reporting on >100 patients with infectious encephalitis, influenza virus has been identified as the (possible) pathogen in 5–9 % of cases (Glaser *et al.*, 2006; Koskiniemi *et al.*, 2001; Rantalaiho *et al.*, 2001). In adulthood, different age groups are equally affected (Rantalaiho *et al.*, 2001).

Clinical syndromes of influenza-associated neurological diseases in adults have not always been clearly defined. Based on the onset of influenza infection in relation to the onset of neurological symptoms, IAE is classified as acute, subacute or late (Akins *et al.*, 2010; Goenka *et al.*, 2014; Steininger *et al.*, 2003). In addition to a classification based on the timing of onset of neurological symptoms, many different neurological disease entities are recognized, which makes classification complex (Akins *et al.*, 2010; Goenka *et al.*, 2014). The absence of distinctive clinical symptoms and validated diagnostic criteria may have resulted in under-recognition of the disease and, possibly, publication bias. This may have influenced the results of our study.

### Pathogenesis

The pathogenesis of acute IAE is not fully elucidated. It appears that both inflammation and viral infection of neurons may cause IAE (Akins *et al.*, 2010; Goenka *et al.*, 2014; Lee *et al.*, 2010; Steininger *et al.*, 2003; Yokota *et al.*, 2000). In the CSF of patients with neurological complications of influenza virus, elevated concentrations of cytokines and anti-influenza antibodies have been reported (Kimura *et al.*, 2008; Lee *et al.*, 2010; Yamashita *et al.*, 2008). Moreover, the presence of avian influenza virus receptors and human influenza virus receptors on neurons, astrocytes and epithelial cells in the brain suggests that these cells are susceptible for infection by influenza virus (Kim *et al.*, 2013; Yao *et al.*, 2008). PCR proven infection of the brain has been reported and the presence of positive-stranded viral RNA in brain neurons indicates active replication of influenza virus in these cells (Gu *et al.*, 2007; Simon *et al.*, 2013).

### Differential diagnosis

The differential diagnosis of acute onset neurological manifestations of influenza virus infection is extensive, and includes both infectious and non-infectious disease entities (Solomon *et al.*, 2012). In approximately half of the cases of apparent infectious encephalitis, an infectious agent was not identified (Koskiniemi *et al.*, 2001; Rantalaiho *et al.*, 2001; Sivertsen & Christensen, 1996; Studahl *et al.*, 1998; Venkatesan *et al.*, 2013).

In the cases we reviewed, many different neurological symptoms were present in the adults with acute onset IAE. Therefore, IAE should be considered in the differential diagnosis of any case of unexplained central nervous system symptoms occurring during the influenza season. Initial work-up in these patients should include cultures/PCR of samples from the nasopharynx/throat and other regions suspected of infection, blood analysis, EEG, MRI, and a cerebrospinal tap (unless signs of increased intracranial pressure are present) (Studahl, 2003). These tests can differentiate infectious from non-infectious disease entities and identify a specific infectious agent.

Brain MRI is the preferred modality in patients with (suspected) encephalitis. It is more sensitive than CT and should be performed in the acute setting when the first neurological symptoms occur in order to detect early cerebral changes associated with neurological manifestations of influenza virus infection or other diseases mimicking this entity (Solomon *et al.*, 2012). Abnormalities on MRI appear as high signal intensity lesions on T2-weighted magnetic resonance images. In general, MRI in patients with neurological complications of influenza virus infection shows lesions in the cerebellum, brain stem, the (splenium of the) corpus callosum or the (bilateral) thalamus (Akins *et al.*, 2010). Subcortical white matter, as well as the cortical and deep grey matter, can be affected. Subclassifications of IAE, based on MRI findings, have been proposed (Akins *et al.*, 2010). We found that a majority of 13/21 (62 %) of the patients had lesions on MRI scans.

The CSF analysis should include opening pressure, white cell count, and glucose and protein concentrations. In addition, molecular detection in CSF for the most common pathogens [herpes simplex virus, varicella zoster virus (VZV), enteroviruses and others depending on the epidemiology] causing infectious encephalitis should be performed. Patient characteristics may necessitate testing of CSF for antibodies or other microorganisms (Solomon *et al.*, 2012). Viral concentrations of influenza virus in the CSF may be too low to be detected by regular diagnostic tests or virus RNA may not be present in the CSF (Akins *et al.*, 2010; Goenka *et al.*, 2014; Lee *et al.*, 2010; Studahl, 2003). In fact, in a single case, influenza virus was detected in brain tissue without detection in CSF (Simon *et al.*, 2013). Testing CSF for influenza virus RNA may, therefore, help to establish IAE, but a negative PCR does not rule out the diagnosis. In the patients we reviewed, influenza virus was detected in CSF in a minority (16 %) of patients.

Detection of intrathecal antibody production may be used to support the diagnosis IAE (Fujimoto *et al.*, 2000; Salonen *et al.*, 1997). The presence of specific IgM or IgA antibodies to influenza virus in CSF without detection of antibodies to other viruses is indicative of intrathecal antibody production, because these antibody classes diffuse into CSF minimally. The Reiber index can be used to determine whether antibodies are produced intrathecally (Reiber & Lange, 1991; Sindic et al, 2001). A CSF/serum pair collected at the same time is required to determine the Reiber index. An index ≥1.5 can be regarded as indicative of intrathecal antibody production; some use a cut-off value of 3 to reduce false positive results. Sequential (paired) samples may be necessary to detect intrathecal antibody production. However, it is unknown how often these antibodies are present in patients with IAE.

An influenza virus infection diagnosis is most frequently based on the detection of influenza virus RNA by PCR of respiratory specimens. Concomitant neurological symptoms may well be associated with this infection, but can also be coincidental. The detection of intrathecal antibody production to influenza virus or the presence of influenza virus in CSF (or brain tissue) provides stronger evidence for a causative role of influenza virus infection. A diagnostic algorithm is presented in Table 2.

**Table 2.** Suggested diagnostic algorithm for IAE

**At initial presentation**	
Collect samples:	Test for:
CSF	PCR for influenza virus
	Antibodies to influenza virus (IgM, IgA, IgG)
	Influenza virus IgG antibody index (with serum)
	Total albumin + albumin index (with serum)
	IgG antibodies to control virus (e.g. VZV)
	IgG antibody index to control virus (with serum)
Serum	Antibodies to influenza virus (IgM, IgA, IgG)
	IgG antibodies to control virus (e.g. VZV)
	Albumin
Throat swab/nasopharyngeal swab	PCR for influenza virus
After 2–3 weeks	
Collect samples:	Test for:
CSF	Antibodies to influenza virus (IgM, IgA, IgG)
	Influenza virus IgG antibody index (with serum)
	Total albumin + albumin index (with serum)
	IgG antibodies to control virus (e.g. VZV)
	IgG antibody index to control virus (with serum)
Serum	Antibodies to influenza virus (IgM, IgA, IgG), new serum paired with serum collected at initial presentation
	Albumin

In our case, three CSF samples were available during the first IAE episode, taken 1, 2 and 10 days after the onset of neurological symptoms. All CSF samples tested negative for influenza virus with PCR. With serology, a low but progressively increasing signal in the influenza IgG test was observed. In the last CSF sample, the Reiber index for influenza A virus IgG was 2.93, which is suggestive for intrathecal antibody production to influenza A virus. In addition, influenza virus RNA was detected in a respiratory sample obtained at day 4 after onset of influenza-like symptoms.

### Prognosis

Data on the prognosis of IAE in adults are scarce. Overall, a full recovery was reported in 61 % of patients that we reviewed. It should be noted though that subtle neurocognitive deficits might have been overlooked in these patients. Studahl (2003) suggested that the results of imaging (either CT or MRI) have a prognostic value in adults with neurological complications of influenza virus infection. Patients without abnormalities on imaging recovered without sequelae in 60 % cases versus 24 % cases when abnormalities were present. In the cases we reviewed, all patients with normal CSF analysis (chemistry and/or PCR) made a full recovery. In the case that a brain MRI was normal, 7/9 (78 %) patients made a full recovery. In contrast, if the MRI was abnormal, only 5/10 (50 %) patients made a full recovery, while 4 (40 %) had sequelae and 1 (10 %) died. Although the numbers are small, CSF analysis and MRI appear to have a prognostic value in patients with acute onset neurological complications of influenza virus infection.

### Prevention and treatment

Influenza vaccination in healthy adults has an efficacy of 59–83 % in preventing influenza infection (Osterholm *et al.*, 2012). Although trials are absent, it is reasonable to assume that vaccination can also prevent neurological complications of influenza virus infection. Only 2/15 (13 %) of patients that we reviewed had received an influenza vaccine. Our patient was not vaccinated either, but now receives yearly influenza vaccination. Influenza vaccination in patients with a history of acute IAE should be considered to prevent subsequent influenza infections.

#### Antiviral agents.

It is unclear whether antiviral drugs have the potential to reduce neurological manifestations of influenza virus infection. Theoretically, in cases of viral invasion of the brain, reducing viral replication could be beneficial. However, it is doubtful whether the drug concentration of the active metabolite oseltamivir carboxylate in CSF is sufficient to inhibit viral replication, since the penetrance of this drug is low (2.9–13 %) (Jhee *et al.*, 2008; Lee *et al.*, 2010). Furthermore, viral RNA was detectable in the CSF of three patients after treatment with oseltamivir for 2, 3 and 5 days with respect to the individual patient, despite treatment with 300 mg day^−1^ in two of these patients (Santini *et al.*, 2012). If IAE is not driven by viral invasion of the brain, but by a ‘cytokine storm’, antiviral treatment may diminish the inflammation caused by influenza infection and thereby help to suppress neurological symptoms. However, data on the exact effects of antiviral drugs in adults with acute onset IAE are lacking.

#### Immunomodulatory therapy.

Since it is assumed that the immune response provoked by influenza virus infection has an important role in the pathogenesis of acute onset IAE, it has been proposed to treat these patients with corticosteroids (Tsai & Baker, 2013). There is limited evidence that treatment with corticosteroids has a positive effect in patients with herpes simplex encephalitis (Kamei *et al.*, 2005; McDaneld *et al.*, 2010). For adults with neurological complications of influenza virus infection, this therapy, as well as treatment with other immunomodulatory medication like alpha interferon and immunoglobulins, has been poorly evaluated (Tsai & Baker, 2013).

#### Treatment in practice.

Our literature survey shows that no standard therapy for neurological complications of influenza virus infection is available. Prescription of a neuraminidase inhibitor and, in certain cases, corticosteroids, is the most-applied treatment. In addition, presenting symptoms may necessitate supportive therapy for vital functions and the use of antiepileptic drugs (Studahl, 2003).

We suggest that initial antimicrobial therapy, started pending the results of diagnostic tests, should consist of acyclovir and broad-spectrum antibiotics aimed at the most prevalent microorganisms causing infectious encephalitis. Oseltamivir may be added to the empiric treatment during the influenza season. Further therapy should be based on patient characteristics and the results of diagnostic tests/pathogenesis of the neurological symptoms (such as the use of corticosteroids when autoimmune or paraneoplastic encephalitis is suspected).

### Conclusions

In conclusion, acute onset IAE in adults is a serious but rare complication of influenza virus infection. It mainly affects immunocompetent males and is not confined to any specific age group in adulthood. Its pathogenesis is not fully elucidated; symptoms may either be the result of an inflammatory response or viral neuro-invasion of the brain. Diagnosing IAE can be difficult, since influenza virus is detected in CSF in a minority of patients. Laboratory detection of influenza virus outside the CNS or serological evidence of a recent infection with influenza virus in combination with brain imaging findings on MRI suggestive of IAE and/or intrathecal antibody production may help to establish the diagnosis. It also requires the exclusion of other potential causative agents. Our diagnostic algorithm may help to uniformly diagnose IAE. Antiviral drugs and corticosteroids are often prescribed to patients with IAE, but their effects are unclear. Approximately 60 % of patients recover without sequelae. A normal CSF analysis and a normal MRI appear to be favourable prognostic factors.

## Supplementary Data

19 Supplementary File 1
